# Converted Macrophage Polarization and Expression of COL6α3—Early Predictors of Remodeling Processes in Adipose Tissue of Male Children

**DOI:** 10.3390/biomedicines13040935

**Published:** 2025-04-10

**Authors:** Robert Mujkić, Darija Šnajder Mujkić, Karla Rožac, Anita Matić, Tanja Kovač Lukić, Dalibor Divković, Kristina Selthofer-Relatić

**Affiliations:** 1 Department of Anatomy, Histology, Embryology, Pathological Anatomy and Pathological Histology, Faculty of Dental Medicine and Health, Josip Juraj Strossmayer University of Osijek, Crkvena 21, 31000 Osijek, Croatia; krozac@fdmz.hr (K.R.); tkovac@fdmz.hr (T.K.L.); 2 Department of Anatomy and Neuroscience, Faculty of Medicine, Josip Juraj Strossmayer University of Osijek, J. Huttlera 4, 31000 Osijek, Croatia; dsnajder@mefos.hr; 3 Clinical Institute of Nuclear Medicine and Radiation Protection, University Hospital Osijek, J. Huttlera 4, 31000 Osijek, Croatia; 4 Department of Pathophysiology, Physiology and Immunology, Faculty of Dental Medicine and Health Osijek, Josip Juraj Strossmayer University of Osijek, Crkvena 21, 31000 Osijek, Croatia; amatic@fdmz.hr; 5 Department of Pediatric Surgery, University Hospital Osijek, J. Huttlera 4, 31000 Osijek, Croatia; divkovicdalibor1369@gmail.com; 6 Department of Surgery, Urology, Orthopedics and Physical and Rehabilitation Medicine, Faculty of Medicine Osijek, Josip Juraj Strossmayer University of Osijek, J. Huttlera 4, 31000 Osijek, Croatia; 7 Department of Cardiovascular Disease, University Hospital Osijek, J. Huttlera 4, 31000 Osijek, Croatia; selthofer.relatic@gmail.com; 8 Department of Pathophysiology, Faculty of Medicine, Josip Juraj Strossmayer University of Osijek, J. Huttlera 4, 3100 Osijek, Croatia

**Keywords:** adipose tissue, obesity, COL6 alpha 3, inflammation, CD163

## Abstract

**Background/Objectives:** Overweight and obesity in early childhood is a serious public health problem as in most cases it persists into adulthood and significantly affects the quality of life. The aim of this study was to investigate the mechanisms that trigger extracellular matrix (ECM) remodeling in the subcutaneous (SAT) and visceral (VAT) adipose tissue of male children in relation to their body weight. **Methods:** During elective abdominal surgery, SAT and VAT were acquired from 75 male subjects undergoing hernia repair (inguinal herniorrhaphy by Ferguson) or orchidopexy. Based on their Z-score, subjects were separated into two groups. The morphometry of both adipose tissue compartments was assessed after hematoxylin and eosin histological staining, immunohistochemistry to quantify CD163^+^ cells and the number of crown-like structures (CLSs), and real-time polymerase chain reaction to assess the relative gene expression for collagen VI subtype alpha 3 (*COL6α3*). **Results:** Obese and overweight individuals were found to have higher numbers of CD163^+^ cells, greater numbers of CLSs in VAT and SAT, and a higher expression of *COL6α3* in both compartments. **Conclusions:** Obesity in childhood may lead to increased *COL6α3* gene expression and promote the activation of macrophage polarization, compromise the structural integrity of the ECM, and thus influence the development of inflammatory processes.

## 1. Introduction

Childhood obesity is today widely recognized as one of the most concerning public health problems of the 21st century [1] because it is often carried over to adulthood, increasing the risk of developing various metabolic disorders that lead to metabolic imbalances [2]. Even more pronounced is the fact that childhood obesity rates equal adult obesity rates, which has led to the emergence of several childhood diseases more appropriate for adulthood, such as dyslipidemia, nonalcoholic fatty liver disease (NAFLD), and the prevalence of type 2 diabetes [3].

For decades, adipose tissue (AT) was considered an inert mass of stored energy with some basic functional properties, such as an insulating function and mechanical support to the structures around which it is located. At birth, subcutaneous (SAT) and visceral (VAT) adipose tissue are already very well developed and account for 16% of the total body mass of a newborn, and by 18 months of age, white adipose tissue (WAT) accounts for approximately 28% of the child’s total body mass [4].

SAT is the most important WAT compartment in lean and healthy individuals and it accounts for more than 80% of the total extent of AT. SAT is the largest repository of WAT, and its importance for energy storage greatly impacts metabolic energy homeostasis. In response and reaction to maintaining a positive energy balance, SAT reorganizes itself by changing the number and size of mature fat cells. SAT acts as a metabolic “sink” for storing excess lipids [5]. Omental, mesenteric, and retroperitoneal adipose tissues are classified as areas credited to VAT. VAT is very metabolically productive and constantly discharges free fatty acids (FFAs) into the bloodstream. The content and amount of VAT contribute greatly to various aspects of metabolic syndrome (MetS), such as dyslipidemia, hyperinsulinemia, systemic inflammation, and atherosclerosis [6,7].

Obesity is characterized by an increased enlargement of adipocytes. Remodeling and reorganization of the extracellular matrix (ECM) are necessary to provide sufficient storage space for the enlarged adipocytes (hypertrophy) and for the formation of new adipocytes through the process of adipogenesis via progenitor cells (hyperplasia). Mature adipocytes store energy in the form of triglycerides, which leads to strong mechanical stress and pressure that is transferred from the outside to the inside of the cells [8,9]. The remodeling of AT involves several processes, including the infiltration of inflammatory cells and the development of an inflammatory response. This response triggers the production of pro-inflammatory cytokines, ultimately leading to structural alterations in AT causing its remodeling [10]. Some researchers use the term “remodeling” to describe the changes that occur in the structural composition of stromal tissue in response to the contraction or enlargement of AT due to fluctuations in food intake. Other researchers, however, use the term “remodeling” to refer to the breakdown and degradation of non-cellular components of AT, such as the ECM, which plays a critical role in shaping the overall architecture of AT [11,12].

The peculiarity of AT is that it contains the highest concentration of type VI collagen when compared to all other tissues [13]. Collagen VI is a non-fibrillar collagen consisting of short triple-helical domains and larger globular domains. In human AT, it has been discovered that AT oxygenation and collagen VI sub-type alpha 3 (*COL6α3*) mRNA are significantly correlated, indicating that individuals with higher *COL6α3* expression have lower AT oxygenation [14], and that lower oxygenation is closely related to the development of the inflammatory process in AT [15].

Saltiel et al. proposed the “phenotypic switching” model in 2007 to explain the correlation between the increased infiltration of adipose tissue macrophages (ATMs) and the exacerbation of inflammation in obesity. The model suggests that obesity changes macrophage polarization [16]. Macrophages are generally considered to represent a heterogeneous population of cells with a variable function that depends on polarization status. Macrophages are described as phagocytic cells that break down and remove cell debris after cell death. This is one of their key functions in fibrogenesis, however, whether fibrosis progresses faster or slower also depends on the type of tissue involved [17,18]. M1 macrophages are characterized as pro-inflammatory phenotypes that have the ability to promote the immune response and exhibit tumoricidal activities, while M2 macrophages exhibit regulatory functions in restoring damaged tissue, remodeling, and promoting the immune response of T-helper type 2 cells [19]. Macrophages in the AT of obese individuals are often clustered around dead adipocytes, which is known as a crown-like structure (CLS) [20], and is one of the commonly recognized hallmarks of low-intensity chronic inflammation [21].

The aim of this study is to investigate the mechanisms of ECM remodeling in different compartments of adipose tissue in male children in comparison with their adipose status.

## 2. Materials and Methods

This study included 75 male subjects between the ages of 2 and 16. These subjects were admitted for elective abdominal surgery (inguinal herniorrhaphy by Ferguson and orchidopexy) at the Department of Pediatric Surgery of the University Hospital Osijek. Informed consent was obtained from all guardians of the subjects involved in the study, and the study was approved by the Ethics Committee of the University Hospital Osijek (No. R2-16 905/2018) and the Ethics Committee of the Faculty of Medicine Osijek, University of Josip Juraj Strossmayer in Osijek (No. 2158-61-07-20-113; Class No. 602-04/20-08/07). Subjects with incarcerated hernias were excluded because of underlying tissue inflammation.

### 2.1. Anthropometric Measurements

Anthropometric measurements included measuring body mass in kilograms (kg), height in centimeters (cm), and waist and thigh circumference in centimeters (cm).

Body height was measured with a standard technique, a tape measure in an upright position, against a wall, without shoes, and facing forward with a deviation of ±0.5 cm. Body mass was determined using a standard technique on a measuring scale before surgery, barefoot, without shoes, and wearing very light clothing with a deviation of ±0.1 kg. Waist and thigh circumferences were measured in centimeters with a tape measure. The waist circumference was determined at the level of the navel area of the abdomen, and the thigh circumference at the mid-thigh.

### 2.2. Body Mass Index and Z-Score

The body mass index (BMI), as one of the most widely used methods of defining obesity, was calculated, is a measure of an individual’s nutritional status and is determined according to the equation given as follows: BMI = body mass (kg)/body height (m^2^).

The Z-score is defined by WHO standard curves, which indicate the number of standard deviations from the median of the population of a normal distribution. A Z-score of +1 indicates risk of overeating, +2 indicates overweight, and +3 indicates obesity [22]. Based on the Z-score, the subjects were divided into two groups: one group with overweight and obesity (n = 33) and another group of subjects with normal body weight (n = 42).

### 2.3. Blood Sample and Serum Analysis

Venous blood samples were collected for biochemical analysis before surgery on an empty stomach, after fasting for at least 12 h, and before an introduction to anesthesia. Biochemical analysis of venous blood serum was performed as part of a regular diagnostic procedure at the Clinical Institute of Laboratory Diagnostics, University Hospital Osijek, where the glucose concentration level and lipid profile (cholesterol, triglycerides, high-density lipoprotein (HDL), and low-density lipoprotein (LDL)) were obtained. Testosterone levels were also measured (MassChrom Steroids in Serum/Plasma Reagent Kit for LC-MS/MS Analysis, Chromsystems Instruments & Chemicals GmbH, Graefelfing, Germany), and values were interpreted using reference tables for steroid hormones provided by the reagent manufacturer.

### 2.4. Subcutaneous and Visceral Adipose Tissue Samples

During the surgical procedure, SAT and VAT samples were taken from the abdominal region. VAT was first collected from the area of the large abdominal sac at the earliest opportunity during surgery (lat. omentum majus), whereas SAT was collected at the end of surgery, immediately before suturing. The samples were then distributed in duplicate into sterile 2 mL plastic cryotubes (Cryovial, Bernard-Pilon, Beloeil, QC, Canada) previously filled with a nontoxic reagent (RNA later, Qiagen, Hilden, Germany) for the preservation and stabilization of human ribonucleic acid and stored at −80 °C until analysis time. The second part of the tissue samples was stored in 2 mL plastic tubes previously filled with 10% buffered formaldehyde (Biognost, Zagreb, Croatia).

The tissue samples were then subjected to the standard procedure for the processing and preparation of histological samples, in which the tissue was dehydrated by immersion in ethanol of increasing concentration for a specified time. After dehydration, the samples were embedded in paraffin blocks and cut to a thickness of 6 µm using a rotary microtome (CUT 4060, SLEE Medical GmbH, Nieder-Olm, Germany) and mounted on special adhesive slides (DAKO, Glostrup, Denmark), which were used for the immunohistochemical analysis (IHC) of adipose tissue.

### 2.5. Adipose Tissue Morphology

To measure the size of adipocytes in SAT and VAT, the samples were dehydrated according to the previously described procedure, cut to a thickness of 6 µm, and stained using the hematoxylin and eosin method. The tissue samples were then examined using a light Zeiss Axioskop 2 MOT microscope (Carl Zeiss Microscopy, Pleasanton, CA, USA), and digital images of the samples were captured using an Olympus DP70 digital camera (Olympus, Tokyo, Japan) connected to the microscope.

**Figure 1:**
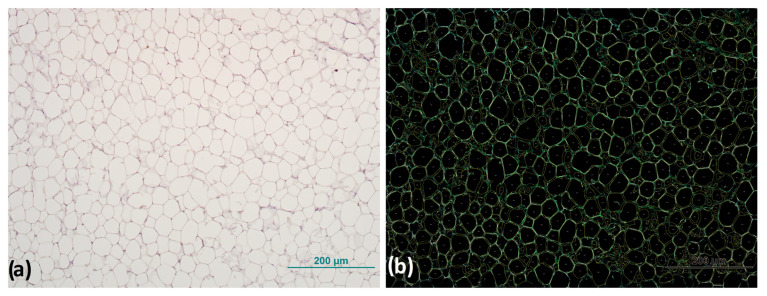
Representative image of adipocyte morphology analysis: (**a**) original image of adipocytes before and (**b**) after processing with analysis software for detection and size measurement of adipocytes. Magnification: 100×. Scale bar 200 µm.

The digital images are stored in an uncompressed file format that ensures high-resolution preservation until they are analyzed. All images were acquired under identical conditions to ensure consistency and minimize any potential variations (magnification 200×, TIFF). Analysis of AT cell size was performed using the free online biological analysis program Fiji v.2.9.0 [23], an ImageJ v.1.54h distribution [24], with the Adiposoft plugin v.1.16 for AT cell size analysis [25]. Before starting the analysis, the parameters of the analysis were set and the possibility of manual correction of the input was set, with the option to exclude all cells that are on the edge or not completely within the frame, “exclude on edges”, and using the default unit of measurement in which the data are displayed at the end of the analysis (Figure 1).

### 2.6. Immunohistochemistry

Immunohistochemistry on all samples of SAT and VAT for CD163^+^ cells was performed at the Clinical Institute of Pathology and Forensic Medicine, University Hospital Osijek, using the fully automated working system BenchMark ULTRA (Ventana Medical systems IBC., Oro Valley, AZ, USA) with rabbit monoclonal primary antibody anti-CD163^+^ (#ab182422, Abcam, Cambridge, UK) at a concentration of 1:80 according to the manufacturer’s instructions. Liver and brain tissues used as positive and negative controls were obtained from the database of the Clinical Institute of Pathology and Forensic Medicine of the University Hospital Osijek, which are used in routine diagnostics.

**Figure 2:**
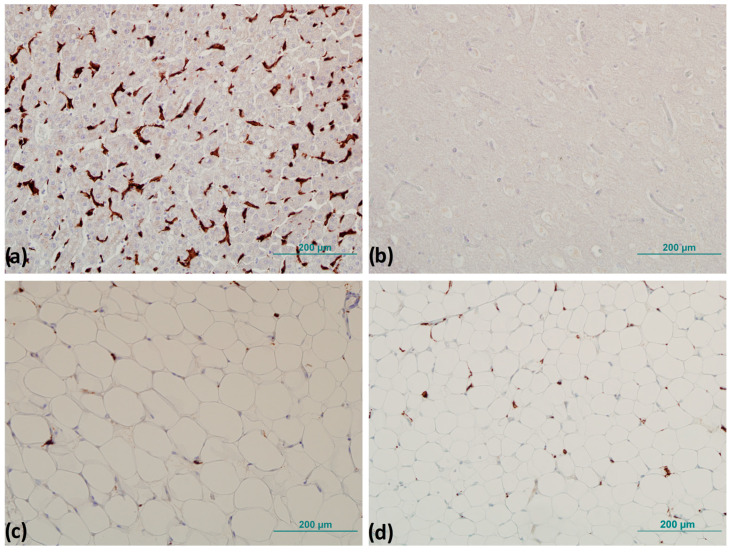
Representative images of immunohistochemistry for positive and negative control and detection of CD163^+^ cells: (**a**) positive control—brown staining (liver tissue) and (**b**) negative control—no browning (brain tissue). CD163^+^ cells detection: (**c**) subcutaneous and (**d**) visceral adipose tissue. Magnification 200×. Scale bar 200 µm.

To precisely identify the number of positive cells after IHC was performed, tissue samples were examined by pathologists on a Zeiss Axioskop 2 light microscope MOT (Carl Zeiss Microscopy, White Plains, NY, USA), on which a histomorphometric grid with an area of 0.17 mm^2^ was attached. The number of CD163^+^ cells was determined by counting the CD163^+^ cells in six randomly selected fields of view under a magnification of 400×, and the mean value of the number of CD163^+^ cells per mm^2^ was determined (Figure 2) [26].

**Figure 3:**
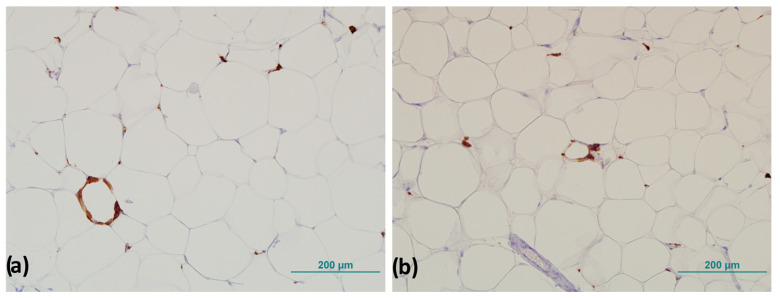
Representative images of crown-like structures in visceral adipose tissue: (**a**) completely formed crown-like structure and (**b**) crown-like structure in forming. Magnification 200×. Scale bar 200 µm.

CLSs were counted throughout the field of view of the entire sample, and the number of CLSs was normalized to the total number of adipocytes according to the method of Fischer et al. described previously (Figure 3) [27].

### 2.7. Determination of Relative Gene Expression

During the surgical procedure, subcutaneous and visceral adipose tissue samples were collected to determine the relative expression of target genes by rtPCR. VAT was collected first at the most feasible time during the surgical procedure, while SAT was collected at the very end of the procedure, immediately before suturing, as described before.

Samples were then distributed in duplicate into sterile plastic 2 mL cryotubes (Cryovial, Bernard-Pilon, Beloeil, QC, Canada), previously filled with RNAlater^®^ solution (Applied Biosystems, Waltham, MA, USA) at a ratio of 1:5 RNAlater^®^, according to the manufacturer’s instructions. RNAlater^®^ solution is a non-toxic reagent that allows for its long-term preservation by penetrating into the tissue and deactivating the RNA-se. Samples stored in RNAlater^®^ were kept at −80 °C until analyzed. The process of sample homogenization and isolation of total RNA from the samples was performed using the reagent TRI (Life Technologies, Carlsbad, CA, USA), according to the standard protocol described by Chomezynski et al. [28].

Sample purity and RNA concentration were tested using a Nanophometer P300 UV/VIS (IMPLEN GmbH, Munich, Germany). The purification of samples and recovery of cDNA was performed according to the manufacturer’s instructions: Sigma-Aldrich (St. Louis, MO, USA) and Applied Biosystems (Waltham, MA, USA). Analyses for the determination of relative gene expression by the rtPCR method were performed in the Laboratory of Immunology, Department of Physiology and Immunology, Faculty of Medicine Osijek, Josip Juraj Strossmayer University of Osijek.

Expression of the collagen 6 subtype-alpha 3 gene (*COL6α3*) was determined (primer sequence: forward 5′-AAGCTCTTAGCCAGCACTCG-3′ and reverse 5′-CACTTTACTGGGGCCGATGT-3′). Low-density lipoprotein receptor related protein 10 (*LRP10*) (primer sequence: forward 5′-CTTCCTACGGGCAGCTCATT-3′ and reverse 5′-CAGGCTCTTGCTCACAGGC-3′) and importin 8 (*IPO8*) (primer sequence: forward 5′-TGTTCAGCTCCTTCCTGATTC-3′ and reverse 5′-CTTCTTACACTTCCACCATAC-3′) were selected for the normalization of gene expression. Both genes were analyzed simultaneously in all samples. Gene expression was normalized to the expression of the *LRP10* gene, which was found to be more stable and equally expressed in all samples. Determination of the gene expression was measured in real-time using an rtPCR instrument (Bio Rad CFX96, BioRad Laboratories Inc., Singapore, Singapore).

### 2.8. Statistical Analysis

The IBM SPSS Statistica (version 21, IBM Corporation, Armonk, NY, USA) program was used for statistical analysis. Data were tested for normality of distribution using the Shapiro–Wilk test. Because there was no deviation from the normal distribution, the results are expressed as mean ± standard deviation, and differences in numerical variables between the two independent groups were tested with the Student’s *t*-test. Correlations were tested with Pearson’s correlation coefficient r. A *p* value of less than 0.05 was considered statistically significant.

## 3. Results

### 3.1. Anthropometric and Laboratory Measurements

The overweight/obese group of boys had a logically higher body mass, BMI, Z-score, and waist and thigh circumference. There was no difference between groups in age and height (Table 1).

In the laboratory measurements, no differences between groups was found in the concentration of total cholesterol, LDL-cholesterol, triglycerides, glucose, and testosterone. The HDL-cholesterol concentration was higher in the group of normal weight children (1.40 ± 0.30 mmol/L) compared to overweight/obese children (1.26 ± 0.29 mmol/L; *p* = 0.040, Student’s *t*-test).

### 3.2. Adipocyte Morphometry

In SAT, the mean adipocyte surface was 1031.15 ± 327.95 µm^2^ in the overweight/obese group, which was significantly higher compared to the adipocyte surface of normal weight male children (572.21 ± 191.95 µm^2^; *p* < 0.001, Student’s *t*-test).

In VAT, the mean adipocyte surface calculated in the overweight/obese group was 407.87 ± 153.69 µm^2^, and in normal weight male children it was 354.52 ± 139.59 µm^2^; therefore, no significant difference between the groups was found (*p =* 0.238, Student’s *t*-test).

The adipocyte surface in SAT correlated positively with body weight (r = 0.378, *p* = 0.010), BMI (r = 0.745, *p* < 0.001), Z-score (r = 0.685, *p* < 0.001), and triglyceride concentration (r = 0.319, *p* = 0.035), and the mean adipocyte surface in VAT correlated positively with body weight (r = 0.339, *p* = 0.023; Pearson correlation).

### 3.3. CD163^+^ Cells and Number of Crown-like Structures

There was a statistically higher number of CD163^+^ cells and CLS in the VAT of the group of overweight male children compared to normal weight subjects, and this difference was also measured when counting the number of CLS in SAT (Table 2).

**Figure 4:**
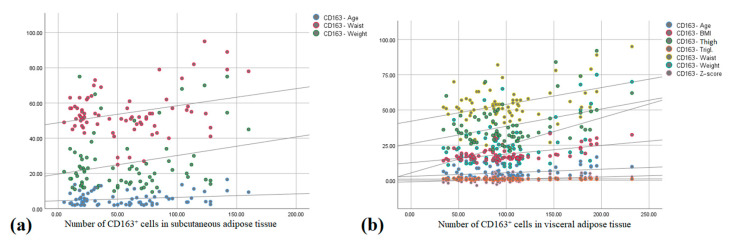
Correlation between the number of CD163^+^ cells in (**a**) subcutaneous adipose tissue and age, waist circumference and weight; and (**b**) visceral adipose tissue and age, BMI, Z-score, thigh and waist circumference, weight, and serum triglyceride concentration (Pearson correlation).

There was a moderate positive correlation between the number of CD163^+^ cells in SAT and age (r = 0.379, *p* = 0.010), body weight (r = 0.304, *p* = 0.042), and waist circumference (r = 0.484, *p* = 0.001) (Figure 4a).

The number of CD163^+^ cells in VAT correlated positively with BMI (r = 0.813, *p* < 0.001), Z-score (r = 0.725, *p* < 0.001), body weight (r = 0.742, *p* < 0.001), thigh (r = 0.532, *p* < 0.001) and waist circumference (r = 0.620, *p* < 0.001), and adipocyte surface in SAT (r = 0.563, *p* < 0.001), and there was a moderate positive correlation between the number of CD163^+^ cells in VAT (r = 0.325, *p* = 0.029), triglyceride serum concentration (r = 0.348, *p* = 0.010), and age (r = 0.379, *p* = 0.010) (Figure 4b).

The number of CLSs in SAT had a moderate positive correlation with Z-score (r = 0.316, *p* = 0.006), BMI (r = 0.372, *p* = 0.001), and adipocyte surface in SAT (r = 0.425, *p* = 0.004), and the number of CLSs in VAT correlated positively with age (r = 0.243, *p* = 0.036), body weight (r = 0.390, *p* = 0.001), Z-score (r = 0.366, *p* = 0.001), BMI (r = 0.517, *p* < 0.001), and adipocyte surface in SAT (r = 0.519, *p* < 0.001) and VAT (r = 0.314, *p* = 0.014; Pearson correlation).

### 3.4. Relative Gene Expression

There was a higher expression of collagen 6 α3 in both SAT and VAT in the group of overweight/obese boys (Table 3).

The expression of collagen 6 α3 correlated positively with the Z-score in both SAT (r = 0.735, *p* = 0.003) and VAT (r = 0.366, *p* = 0.001; Pearson correlation).

## 4. Discussion

This study is another step in the research of obesity and metabolic-related diseases caused directly or indirectly by obesity and overweight, which are an increasing problem not only for the elderly population but for all age groups. In the group of overweight and obese children, we found a considerably lower concentration of HDL cholesterol when compared to the group of subjects with normal body weight. One of the characteristics of obesity is dyslipidemia, which is characterized by increased FFA, decreased HDL, and normal or slightly increased LDL levels [29]. Obesity not only affects HDL concentration but also its functionality, and recent evidence suggests that HDL may lose its protective activity and become atherogenic under certain conditions [30]. Studies in animal models have shown that the inhibition of cholesteryl ester transfer protein blocks and affects the exchange between triglycerides and cholesterol, leading to an increase in HDL levels [31].

We did not find a significant alteration in triglycerides or LDL levels, but we found statistical significance for HDL levels, which may indicate the beginning of a protective mechanism in normal weight individuals. Linsel-Nitschke et al. noted that genetic factors primarily dictate the levels of LDL cholesterol, but this is not the case for triglycerides, which can be significantly influenced by environmental factors such as a sedentary lifestyle or individual age [32]. Controlling these risk factors in childhood could be very useful in preventing the development of more severe metabolic diseases in adolescence and old age.

Analysis of the relationship between certain parameters revealed a positive correlation between triglycerides and the adipocyte surface area in SAT.

Previous studies have shown that larger adipocytes accumulate much more triglycerides and release more FFA than smaller adipocytes [33]. There is also a positive correlation when comparing triglyceride levels with the number of CD163^+^ cells in VAT. In the study of diet-induced obesity, it was confirmed that macrophages in adipose tissue metabolically activate FFAs released by the lipolysis of triglycerides from adipocytes or triglyceride-rich lipoproteins by lipoprotein lipase involving the Cd36 receptor. In an obese mouse model, Cd36 was found to be a class B scavenger receptor and to function as a fatty acid transporter by promoting increased fatty acid uptake. It is considered to play the role of a mediator in communication between adipocytes and macrophages and it stimulates the secretion of adipocytokines from macrophages [34,35]. No significant difference in glucose levels between groups was found in this study. One of the reasons for this could be that the metabolic profile is more homogeneous in obese children who have not yet reached puberty [36], and this hypothesis should perhaps be investigated further by another study.

In the SAT of overweight and obese subjects, the surface area of adipocytes was significantly higher compared with the surface area of adipocytes in the group of subjects with normal body weight.

Studies in animal models show that adipogenesis in SAT occurs early in embryogenesis and that the number of adipocytes remains stable after birth, whereas adipocytes in VAT only differentiate postnatally [37]. In humans, SAT is formed from 14 to 24 weeks of gestation, extending from the head and neck to the caudal area [38], and the number of adipocytes remains largely stable after the first year of life until adolescence [39]. This may be the reason that we only found changes in adipocyte size in SAT. On the other hand, VAT remains reduced and unchanged until adolescence [40,41], and this may be one of the reasons that we only found changes in adipocyte surface area in SAT, suggesting that cellular hypertrophy in early age is present only in SAT and not in VAT.

A positive correlation was found between subcutaneous adipocyte surface area and body weight, BMI, Z-score, and serum triglyceride concentration, and similar results were confirmed by Kruchen et al. [42]. Other authors have also reported larger adipocytes in SAT in obese children, suggesting that hypertrophy and hyperplasia begin at a young age [43,44]. The results of the study by Arner et al. show that individuals with adipocyte hypertrophy have a significantly less favorable metabolic profile than those with hyperplasia [45]. There are also differences in the morphology of adipocytes in different adipose tissue depots. In their study, Tarabra et al. showed that adipocytes collected from omentum VAT were much smaller, more numerous, and had greater lipolytic activity than adipocytes from SAT collected from the abdominal area of obese adolescents aged 16 to 22 years [46]. In our study, the measured area of VAT adipocytes was smaller compared to SAT adipocytes. Our results are also confirmed by the study of Landgraf et al. performed on children, in which they found similar correlations between normal weight and obese subjects [47].

The number of CD163^+^ cells was significantly higher in the VAT of the group of overweight and obese subjects in this study. A lower number of CD163^+^ cells was present in the SAT of subjects with normal body weight, but there was no significant difference when comparing the two groups of subjects. In liver diseases characterized by an inflammatory process and the appearance of fibrosis, a strong increase in the number of macrophages has been recorded, and an increase in macrophage number has also been found in obese children who have a high pediatric index for the development of NAFLD fibrosis [48,49]. Characteristically, the amount of adipose tissue in the liver is closely related to the degree of obesity but is much more common in obese people diagnosed with type 2 diabetes than in people with the same body mass index but who do not have type 2 diabetes [50].

In our study, the number of CD163^+^ cells in VAT was higher in the group of overweight and obese subjects and correlated positively with age, BMI, and Z-score. CD163 is expressed exclusively in monocytes (low expression) and macrophages (high expression), is often used as a M2 marker, and is upregulated in several chronic inflammatory diseases such as atherosclerosis, chronic heart failure, and diabetes mellitus [51]. Because an increase in CD163^+^ macrophages happens in tissue during the progression of inflammation, this could also explain the increase in CD163^+^ cells in the adipose tissue of our overweight group of children.

The number of CLSs in SAT and VAT was significantly increased in the group of overweight and obese subjects and correlated positively with BMI and the adipocyte surface area in SAT; additionally, the number of CLSs in VAT correlated positively with the age, body mass, and adipocyte surface area of both AT compartments.

Our results support previous findings of an increased presence of CLSs in SAT and VAT in overweight and obese individuals [47,52]. This may suggest that low-intensity inflammation is also characteristic for children with obesity compared to non-obese children. In our study, the number of CLSs was increased in SAT and VAT in overweight and obese subjects, but a greater number of CLSs was present in VAT, whereas the study conducted by Murano et al. [53] showed the presence of CLSs in a much greater number exclusively in VAT in animal models, which is similar to our results. One of the reasons why more CLSs accumulate in VAT may be that adipocytes in VAT reach a “critical point” much earlier, at which they can undergo the process of hypertrophy that leads to much earlier cell decay than adipocytes in SAT [40,53]. Although the number of CD163^+^ cells was only higher in VAT, an increased presence of CLSs was noted in both SAT and VAT, so the inflammatory process could be less pronounced in SAT although its remodeling had started to be shown by the CLSs.

A significantly higher expression of *COL6α3* was detected in both AT depots in the overweight and obese group compared with normal body weight subjects. A statistically significant positive correlation was found between *COL6α3* expression in VAT and Z-values, and this association was also found in SAT. Adipocytes are embedded in the ECM, which allows for AT expansion, provides mechanical support, and is involved in the conduction of signaling pathways between cells. *COL6α3* is an essential component of the AT ECM, and its absence results in an uncontrolled expansion of individual adipocytes and is paradoxically associated with a marked improvement in energy homeostasis [54]. Fibrillar *COL6α3* in AT is closely associated with obesity, inflammation, insulin resistance (IR), and cancer. Spencer et al. state in their study that the occurrence of fibrosis and increased *COL6α3* expression in SAT is strongly correlated with BMI [55]. Pasarica et al. also confirmed that *COL6α3* mRNA levels in SAT positively correlate with BMI and total body fat mass in obese adults [56]. In our study, we obtained the same results in children, with a positive correlation of *COL6α3* expression with Z-values in the group of overweight and obese subjects in both SAT and VAT, whereas Spencer and Pasarica et al. analyzed only SAT. The degree of fibrosis itself is associated with the development of IR, while fibrous areas contain more macrophages. Other studies confirm these conclusions. Tam et al. suggest in their research on children that a higher expression of inflammatory genes and other inflammation markers can be used as a predictive tool in young subjects for early stages of adipose tissue transformation from physiologically healthy to pathological [57]. Dankel et al., in their study, shared the insight that elevated *COL6α3* expression in the adipocytes of adults has been linked to IR, with expression levels correlating positively with BMI and markers of metabolic dysfunction [15].

A study performed in animal models using COL6KO mice suggests that the ECM can severely restrict adipocyte expansion, leading to adipocyte necrosis and the formation of CLS [54]. Our results confirm and follow the above theories and the presented results of the previously mentioned studies stating that *COL6α3* can directly affect the limitation of fat storage [58]. Although a direct relationship with low-intensity inflammation has not been fully elucidated, two separate studies by Pasarica et al. and Hosogai et al. suggest that hypoxia and decreased capillary density resulting from excessive fibrosis in AT may promote macrophage chemotaxis and the development of AT inflammation [14,59].

Interestingly, we found higher numbers of CD163^+^ cells in the VAT of obese children, which could also be due to a higher expression of pro-inflammatory adipocytokines such as TNF-α, with which *COL6α3* gene expression positively correlates, as was reported in the study by Wang et al. [60]. Evidence of dysfunction in the process of AT remodeling due to increased or decreased nutritional intake is one of the main characteristics of obesity associated with the onset of the MetS. The mechanisms that are involved in the expansion of AT in obesity are not completely consistent, and the patterns of AT storage and expansion vary from person to person. These differences in the methods and mechanism of AT storage among individuals lead to differences in the development and incidence of metabolic disease among people with the same degree of obesity [61,62]. It is also thought that a greater deposition of certain types of collagens leads to the appearance of fibrosis in the ECM and limits the further expansion and development of AT [9].

Our preliminary study, which included male children and adolescents in a smaller number [63], confirmed these findings, which leads us to suggest that the pathological nature of fibrosis formation and the onset of the pathological ECM remodeling process may be related to the type of collagen rather than the extent of fibrosis itself. In AT, the ECM undergoes constant remodeling that allows adipocytes to grow or shrink normally depending on nutritional intake [64]. However, in obese individuals, the excessive accumulation and deposition of collagen in the ECM promotes the formation of fibrosis and reduces elasticity, preventing further healthy expansion of AT and causing the appearance of IR and various metabolic diseases [65].

## 5. Conclusions

In male children, obesity can lead to subcutaneous adipocyte hypertrophy, increased *COL6α3* expression in SAT and VAT, and promote changes in macrophage polarization that affect their accumulation. The altered adipose tissue environment associated with obesity disrupts the balance and composition of the ECM, which may compromise its integrity and lead to the development of inflammatory processes.

The dynamics of these processes, captured at a young age, will further elucidate the processes of the conversion of physiologic to pathologic adipose tissue, which will contribute to a better understanding of the occurrence of early metabolic syndrome symptoms in children with obesity in their early childhood and adolescence.

## Figures and Tables

**Table 1:** Anthropometric measurements of the two groups of subjects.

	Normal Body Weight	Overweight/Obese	*p* *
n	42	33	
Age (years)	5.08 ± 3.17	6.24 ± 3.85	0.157
Body weight (kg)	20.31 ± 9.61	32.35 ± 20.07	0.001
Height (cm)	110.76 ± 21.71	114.73 ± 30.16	0.510
BMI (kg/m^2^)	19.75 ± 1.43	26.72 ± 5.18	<0.001
Z-score	0.40 ± 1.00	2.36 ± 1.49	<0.001
Waist circumference (cm)	50.65 ± 10.97	58.61 ± 13.83	0.008
Thigh circumference (cm)	34.74 ± 11.63	43.71 ± 14.96	0.005

Results are displayed as mean ± standard deviation; * Student’s *t*-test.

**Table 2:** Number of CD163^+^ cells and crown-like structures.

	Normal Body Weight	Overweight/Obese	*p* *
n	42	33	
CD163^+^ cells			
SAT	73.36 ± 34.26	84.29 ± 34.60	0.403
VAT	98.57 ± 20.74	168.18 ± 33.13	<0.001
Crown-like structures			
SAT	0.50 ± 0.70	1.12 ± 1.08	0.004
VAT	2.93 ± 1.73	4.36 ± 2.32	0.003

Results are displayed as mean ± standard deviation; * Student’s *t*-test; SAT—subcutaneous adipose tissue, VAT—visceral adipose tissue.

**Table 3:** Relative mRNA expression of collagen 6 α3 in subcutaneous and visceral adipose tissue between groups of boys.

	Normal Body Weight	Overweight/Obese	*p* *
Collagen 6 α3 in SAT	0.83 ± 0.15	1.14 ± 0.18	0.001
Collagen 6 α3 in VAT	0.68 ± 0.04	0.84 ± 0.08	<0.001

Results are displayed as mean ± standard deviation; * Student’s *t*-test; SAT—subcutaneous adipose tissue, VAT—visceral adipose tissue.

## Data Availability

The original contributions presented in this study are included in the article. Further inquiries can be directed to the corresponding author.

## Abbreviations

The following abbreviations are used in this manuscript:

NAFLD	Nonalcoholic fatty liver disease
AT	Adipose tissue
SAT	Subcutaneous adipose tissue
VAT	Visceral adipose tissue
WAT	White adipose tissue
FFAs	Free fatty acids
MetS	Metabolic syndrome
ECM	Extracellular matrix
COL6α3	Collagen VI subtype alpha 3
ATMs	Adipose tissue macrophages
CLS	Crown-like structure
BMI	Body mass index
HDL	High density lipoprotein
LDL	Low density lipoprotein
IHC	Immunohistochemistry
LRP10	Low-density lipoprotein receptor related protein 10
IPO8	Importin 8
IR	Insulin resistance
